# Genetics of Alzheimer’s Disease in the African American Population

**DOI:** 10.3390/jcm12165189

**Published:** 2023-08-09

**Authors:** Mark W. Logue, Shoumita Dasgupta, Lindsay A. Farrer

**Affiliations:** 1 National Center for PTSD, Behavioral Sciences Division, VA Boston Healthcare System, Boston, MA 02130, USA; loguem@bu.edu; 2 Department of Psychiatry, Boston University Chobanian & Avedisian School of Medicine, Boston, MA 02118, USA; 3 Department of Medicine (Biomedical Genetics), Boston University Chobanian & Avedisian School of Medicine, Boston, MA 02118, USA; dasgupta@bu.edu; 4 Department of Biostatistics, Boston University School of Public Health, Boston, MA 02118, USA; 5 Department of Medical Sciences and Education, Boston University Chobanian & Avedisian School of Medicine, Boston, MA 02118, USA; 6 Department of Neurology, Boston University Chobanian & Avedisian School of Medicine, Boston, MA 02118, USA; 7 Department of Ophthalmology, Boston University Chobanian & Avedisian School of Medicine, Boston, MA 02118, USA; 8 Department of Epidemiology, Boston University School of Public Health, Boston, MA 02118, USA; 9 Alzheimer’s Disease Research Center, Boston University Chobanian & Avedisian School of Medicine, Boston, MA 02118, USA

**Keywords:** Alzheimer disease, African American, genetics, genome-wide association studies, candidate gene studies, genomic/exomic sequencing, *APOE*, *AKAP9*, polygenic risk score

## Abstract

Black/African American (AA) individuals have a higher risk of Alzheimer’s disease (AD) than White non-Hispanic persons of European ancestry (EUR) for reasons that may include economic disparities, cardiovascular health, quality of education, and biases in the methods used to diagnose AD. AD is also heritable, and some of the differences in risk may be due to genetics. Many AD-associated variants have been identified by candidate gene studies, genome-wide association studies (GWAS), and genome-sequencing studies. However, most of these studies have been performed using EUR cohorts. In this paper, we review the genetics of AD and AD-related traits in AA individuals. Importantly, studies of genetic risk factors in AA cohorts can elucidate the molecular mechanisms underlying AD risk in AA and other populations. In fact, such studies are essential to enable reliable precision medicine approaches in persons with considerable African ancestry. Furthermore, genetic studies of AA cohorts allow exploration of the ways the impact of genes can vary by ancestry, culture, and economic and environmental disparities. They have yielded important gains in our knowledge of AD genetics, and increasing AA individual representation within genetic studies should remain a priority for inclusive genetic study design.

## 1. Introduction

Alzheimer’s disease (AD) is the most common form of dementia, comprising 60–80% of dementia cases [1]. There are environmental influences and health conditions that impact the rate of AD, including education [2], psychopathology, obesity and vascular-related health factors, trauma exposure, social engagement [3], and head injury. AD is approximately twice as prevalent in Black/African American (AA) individuals compared to White non-Hispanic/European ancestry (EUR) individuals [4,5,6]. The reasons for this disparity in risk are not fully understood [7]. For example, the prevalence of AD in AA individuals may be inflated due to biases in the methods for cognitive testing, which may systematically yield lower ratings in AA seniors [8,9]. The AA population also carries a higher burden of poor cardiovascular health [10] and diabetes [11] which are AD risk factors. Differences in education quality and environmental stressors related to economic disparities likely also contribute to the increased risk of AD in AA individuals [12,13,14]. In addition, some of the differences in AD risk may be due to genetic factors, which are the focus of this review.

In this review, we discuss findings from studies of individuals who self-reported as Black or AA, as well as those identified via cluster analysis of genetic profiles to be primarily of African ancestry. Most of these studies included persons living in the United States (US) and thus in similar Westernized social contexts. We also refer to genetic results obtained from African and other African ancestry populations where relevant. However, we acknowledge that both racial classifications and genetically clustered ancestry groups are poor proxies for biological variability [15,16,17]. Moreover, both self-reported race and genetic ancestry are correlated with many social determinants of health (SDOH), which also have a substantial influence on AD risk, presentation, and detection [18]. In the US, groups that differ by ancestry also will differ based on their life history, environment, opportunity, and exposure to systemic biases (e.g., [19]). Therefore, it is important to acknowledge that differences observed between studies in AA cohorts and EUR cohorts are not necessarily due to differences in the effects of specific variants but rather may represent a complex interaction between SDOH and genetics.

Currently, EUR cohorts are vastly overrepresented in genetic studies compared to other ancestry groups. For example, Sirugo et al. [20] found that over 50% of genome-wide association studies (GWAS) were focused on EUR cohorts, and 80% of participants in GWAS were primarily EUR. This difference limits our understanding of the genetic basis of AD in non-EUR populations because the impact of particular genetic loci on AD can vary by ancestry [21]. There are several reasons for this variability, including, but not limited to, ancestry-specific risk variants (alleles), different patterns of linkage disequilibrium (LD, i.e., correlations among proximal genetic variants), and modifying effects of AD risk variants by other genes and environmental factors.

Epidemiological studies comparing the risk of AD in EUR and AA families have found important differences and commonalities. Compared to individuals without AD, relatives of AD cases had an overall 1.5 times increased risk of AD in both AA and EUR families [22]. Green et al. compared the risk of AD in first-degree relatives of EUR and AA AD cases enrolled in the Multi-Institutional Research on Alzheimer Genetic Epidemiology (MIRAGE) Study to the risk for their spouses [23]. This comparison is informative because AD cases and spouses presumably also share many of the environmental risk factors for AD but none of the genetic risk factors. They found that the proportional increase of risk in first-degree relatives was approximately 2.5 times the risk in spouses of both EUR and AA cases. Therefore, although AA participants had a higher rate of AD overall, the relative increase due to genetics and possibly shared environment was consistent between AA and EUR subjects. Another study of MIRAGE participants showed a protective effect of education and a deleterious effect of head injury of similar magnitude in AA and EUR individuals [24]. A similar protective effect of alcohol use in both groups and no significant effect of smoking in either group were also observed, although caution should be taken when interpreting estimates of the effects of alcohol and smoking due to potential biases [25,26,27].

## 2. Identification of AD Risk Genes in EUR Cohorts

Most of the genes implicated in AD were identified in studies of EUR cohorts. Some early-onset (before age 60) cases of familial AD, specifically those that display an autosomal dominant inheritance pattern, are associated with rare, highly penetrant mutations primarily in the presenilin (*PSEN1* and *PSEN2*) and amyloid precursor protein (*APP*) genes. In contrast, the genetic risk of the more common “late-onset” form of AD is determined by many genetic loci. The gene with the largest effect on AD risk, *APOE*, has three major isoform variants (alleles). Compared to the most common ε3 allele, the ε4 allele increases AD risk in a dose-dependent fashion [28] with odds ratios (ORs) of 3.2 and 14.9 for the ε3/ε4 and ε4/ε4 genotypes, respectively relative to the ε3/ε3 genotype [29]. In contrast, the ε2 allele is protective against AD with ORs of approximately 0.6 and 0.5 for the ε2/ε3 and ε2/ε2 genotypes, respectively [29,30]. However, the ε4 and ε2 effects appear much stronger among neuropathologically confirmed AD cases and controls with ORs of 31.2 for the ε4/ε4 genotype and 0.13 for the ε2/ε2 genotype [30]. Many other loci have been associated with AD in EUR GWAS using genome-wide genotyping arrays, which measure as many as several million single nucleotide polymorphisms (SNPs, i.e., single base-pair changes), and many million SNPs whose genotypes were imputed from the genotype array data using large population reference panels [31,32,33,34]. The most recent and largest of these studies included approximately 85,000 diagnosed AD cases and 296,000 controls and identified 75 independent AD risk loci [33]. These loci have a much lower impact than *APOE* on AD risk because the effect alleles are either rare or have smaller effect sizes. Most identified common (minor allele frequency [MAF] > 1%) variants have ORs in the range of 0.8 to 1.2. Higher ORs were observed with rare variants (MAF < 1%). For example, the *TREM2* R47H missense mutation (rs75932628), which was initially identified as an AD risk variant in EUR cohorts by exome sequencing [35,36], has a MAF of 0.003 and an OR of 2.39 [33]. 

Improvements in the coverage of genome-wide genotyping arrays and multi-population whole genome sequence reference panels used for variant imputation have increased the accuracy and expanded the lower boundary of MAF of GWAS analyses, and indeed, several associations have been identified for variants with MAFs as small as 1%. However, analysis of genotypes derived from DNA sequence data remains the gold standard for assessing the impact of rare variants (MAF < 1%) on disease, and sequencing remains the only way to observe ultra-rare variants, which may be limited to a single family or a few individuals. Initial whole exome sequencing (WES) studies of relatively small samples containing several thousand individuals identified associations with missense variants in *TREM2* and *PLD3* [35,36,37]. Many additional rare variant associations were discovered by WES of much larger samples comprised of more than 10,000 individuals. Bis et al. conducted a genome-wide scan for AD using WES data obtained from a primarily EUR discovery cohort of 5740 cases and 5096 controls enrolled in the Alzheimer’s Disease Sequencing Project (ADSP) and several EUR replication cohorts that collectively included 12,121 cases and 18,789 controls who were assayed with a mix of WES and chip-based genotyping [38]. Genome-wide significant (GWS) associations were observed with variants in previously established AD loci, including *TREM2, PILRA, MS46A*, and multiple genes in the *APOE* region. GWS association was also identified with a rare variant in the non-coding RNA *AC099552.4* and several variants in *IGHG3*. Gene-based tests, including only rare variants, implicated known AD risk genes, including *ABCA7* and *TREM2*, as well as a novel gene *ZNF655*. Another ADSP study focusing on a subset of 507 genetically enriched EUR AD cases detected GWS association with a rare missense variant in *CASP7* that encodes a caspase protein known to cleave a toxic fragment from the amyloid precursor protein [39]. Analyses of the ADSP WES dataset stratified by *APOE* ε4 carrier status yielded a novel GWS association of AD with a variant in *GPAA1* among persons lacking ε4 and study-wide significant gene-based associations with *OR8G5, IGHV3-7*, and *SLC24A3* among ε4 carriers [40]. Recently, Holstege et al. identified novel associations with rare predicted-damaging variants in *ATP8B4* and *ABCA1* in a WES study of 16,036 AD cases and 16,522 controls [41]. 

Collectively, the genes identified in these studies implicated the involvement of many biological pathways in AD pathogenesis, including misprocessing of amyloid-B and Tau, lipid processing and transport, neuroinflammation, immune function, neuronal development, and intracellular vesicular trafficking.

## 3. Association of AD with APOE Genotype in African American Cohorts

The *APOE* ε4 and ε2 alleles are more frequent in persons of African than European ancestry [42,43], but the estimated effect sizes for ε4 and ε2 differ between these groups. Early studies of the AD/*APOE* association conducted in relatively small AA cohorts yielded inconsistent findings with respect to the effect of ε4 heterozygosity on AD risk. Some studies reported a significantly increased risk of AD among ε3/ε4 compared to ε3/ε3 subjects with a smaller effect size than that observed in EUR cohorts (OR = 2.3–2.6 in AA cohorts versus > 3.0 in EUR cohorts) [29,30,44,45], but other studies did not observe a significantly elevated risk for AA ε4 heterozygotes [29,45,46]. Most studies found evidence of a deleterious effect of ε4 homozygosity, but the effect sizes varied substantially, with ORs ranging from 3.0 to 10.5 [29,45,46,47]. The protective effect of ε2 in AA cohorts has been confirmed in some studies (OR~0.42 for participants with ε2/ε2 and ε2/ε3 genotypes vs. those with ε3/ε3 [45,48] and OR = 0.41 for ε2/ε3 vs. ε3/ε3 [44]) but not in others [46]. Interestingly, ε2 OR estimates are consistently lower (0.41–0.43) in AA cohorts than those observed in EUR cohorts (OR~0.6; Table 1).

There is some evidence that the combination of *APOE* ε4 and exposure to environmental risk factors further increases AD risk. Recently, a large-scale examination of AD and related dementias (ADRD), *APOE* ε4, posttraumatic stress disorder (PTSD), and traumatic brain injury (TBI) in US veterans from the Million Veteran Program (MVP) observed that ε4, PTSD, and TBI were all major ADRD risk factors [49]. It was also found that the increased AD prevalence associated with TBI in an MVP AA cohort was further increased progressively among ε4 heterozygotes and homozygotes and that this increase was larger in the AA cohort than in the EUR cohort. 

Several lines of evidence suggest that there might be multiple factors contributing to the lower impact of ε4 on AD risk in AA individuals compared to those in other populations. It has been shown that the AD/ε4 association is weaker in Yorubas from Ibadan, Nigeria, compared to AA individuals [45,50,51], suggesting a modification of the ε4 effect by genetic factors in European or other populations that are present in AA individuals due to admixture or variations in exposures and lifestyle [52]. 

Multiple studies have implicated other variants in the *APOE* region as an explanation for population differences in the magnitude of the effect of ε4 on AD risk. Rajabli et al. [53] examined the effect of genetic ancestry on the AD/*APOE* association in AA (1766 AD cases and 3730 controls) and Puerto Rican (220 AD cases and 169 controls) participants. Whereas the overall proportion of African ancestry had no effect on the pathogenicity of ε4 in either cohort, they found that the ε4 allele on an African-ancestry background haplotype (profile of variants in the immediate chromosomal region containing the two SNPs that determine the *APOE* isoform alleles) conferred less risk than ε4 present on a EUR haplotype background (OR_AA_ = 2.34 vs. OR_EUR_ = 3.05, *p* = 0.005). This pattern was also observed in an analysis of African-ancestry and EUR ε4 haplotypes in a Puerto Rican sample (OR_AA_ = 1.26 vs. OR_EUR_ = 4.49, *p* = 0.019). Recently, Le Guen et al. [54] identified an *APOE* coding variant, R145C (rs769455), which is very rare in non-African ancestry populations and occurs only on the haplotype background containing the ε3 allele. Stratified analyses in a discovery AA cohort of 2888 cases and 4957 controls indicated that R145C was associated with AD among ε3/ε4 heterozygotes (OR = 3.01, *p* = 6.0 × 10^−6^) but not among those with the ε2/ε3 or ε3/ε3 genotypes. Moreover, ε3/ε4 individuals with the R145C mutation had an earlier age of onset (*p* = 3.4 × 10^−6^). 

Based on the observation that the ε4/AD association is progressively weaker in East Asian (EASN), EUR, and AA populations, respectively, Choi et al. [55] examined SNPs in the *APOE* region that showed a successive increase or decrease in allele frequency among 19,398 EASN individuals including Korean and Japanese participants, 15,836 EUR individuals, and 4985 AA individuals. Only one SNP, rs405509, located in the *APOE* promoter, showed a consistently large difference in genotype frequencies across populations that could account for the observed variability in the magnitude of the effect of ε4 on AD risk. Among those with the ε4/ε4 genotype, AD risk increased substantially in a dose-dependent manner with the number of rs405509 T alleles in the EASN (TT: OR = 27.02, *p* = 8.80 × 10^−94^; GT: OR = 15.87, *p* = 2.62 × 10^−9^) and EUR (TT: OR = 18.13, *p* = 2.69 × 10^−108^; GT: OR = 12.63, *p* = 3.44 × 10^−64^) groups and rs405509-T homozygotes had a younger onset and more severe cortical atrophy than those with G-allele. This association could not be evaluated in the AA cohort due to small samples for two rs405509 genotypes within *APOE* genotype subgroups. Subsequent functional experiments using *APOE* promoter fragments demonstrated that TT lowered *APOE* expression in human brain tissue and serum. These results suggest that the modifying effect of the rs405509 genotype explained much of the inter-population variability in the AD/ε4 association.

In summary, the *APOE* ε4 allele represents the largest single AD genetic risk factor in AA individuals, although the effect is attenuated relative to the risk observed in EUR cohorts. Multiple investigations have indicated that at least some of the differences across populations are due to local ancestry and other variants in the *APOE* region. Recent research in AA cohorts has also demonstrated that *APOE* ε4, in combination with environmental exposures, including TBI, worsens the impact of the exposures. A thorough understanding of the impact of the *APOE* locus is at the forefront of AD research because of its linkages to ancestry, environmental factors which may differ as a function of cultural and systematic biases, and the high impact of the *APOE* alleles on AD risk which makes it a compelling therapeutic target. 

## 4. Candidate Gene Studies in African American Cohorts

Candidate gene studies examine the association with variants or genes based on a priori hypotheses about the role that they might have in disease pathogenesis. Several associations have been observed in candidate gene studies of AA cohorts (Table 2), which are briefly described here. Erlich et al. [56] investigated a cluster of *PON* genes, which have a role in lipid processing and are associated with cardiovascular disorders [57,58], in EUR and AA families from the MIRAGE Study. In the AA families, they found eight variants associated with AD risk, the strongest of which was rs987539 in *PON2* (*p*~0.0001). Recognizing that vascular and inflammatory factors contribute to AD risk and the role of *NOS* genes in these pathways [59,60], Akomolafe et al. [61] evaluated the association of AD with the endothelial nitric oxide synthase (*NOS3*) Glu298Asp variant (rs1799983) and 10 other *NOS* variants in AA and EUR AD cases and controls from the MIRAGE Study and found a study-wide significant association with rs1799983 in the AA (*p* = 0.002) but not the EUR sample. 

Studies of African ancestry cohorts, including AA and Caribbean-Hispanic individuals, contributed to the robust findings that dysregulation of cellular vesicular trafficking is an important pathway leading to AD. Motivated by the role of protein sorting genes in Aβ accumulation, Rogaeva et al. [62] examined the association of variants in members of the vacuolar protein sorting 10 (Vps10) domain-containing receptor protein family including the sortilin-related receptor (*SORL1*) gene in a multi-ancestry sample of EUR, AA, Caribbean Hispanic, and Israeli-Arab cohorts. They identified associations of AD with multiple *SORL1* haplotypes, several of which were trans-ethnic and others specific to a single group. A haplotype comprised of three SNPs towards the 3′ end of *SORL1* was nominally associated with AD (*p* = 0.0025 with ACT haplotype derived from rs3824968, rs2282649, and rs1010159) in the AA cohort, while in the Caribbean-Hispanic cohort, association was observed with a haplotype of three SNPs located near the 5′ end of the gene (*p* = 0.0053 with the CGC haplotype derived from rs668387, rs689021, and rs641120). These findings and other experiments from that study suggest that the associated variants impact AD risk through their effects on APP sorting and cleavage. 

**Table 2:** Significant genetic associations for AD risk and AD-related traits in African American cohorts.

Study Type	Source	African American Cohort Characteristics	Gene	Variant/ Trait	Ref/Alt Allele	RefAF AFR/ EUR *	Effect Size (OR or β)	Uncorrected *p*-Value
AD Candidate Gene (most significant locus reported)	Erlich et al. 2006 [56]	Family cohort of 243 cases and 224 controls	*PON2*	rs987539	C/T	29%/50%	NA	~0.0001
	Akomolafe et al. 2006 [61]	Family cohort of 241 cases and 226 controls.	*NOS3*	Glu298Asp (rs1799983)	G/T	93%/66%	NA	0.002
	Reitz et al. 2011 [63]	Family cohort of 310 cases and 327 controls	*SORCS1*	rs1887635	G/A	90%/80%	β = 0.30	0.053
	Vardarajan et al. 2012 [64]	Family cohort of 513 cases 504 controls	*KIAA1033*	rs1196806	A/G	7%/15%	OR = 1.5	0.03
	Janicki et al. 2013. [65]	Prospective Case/control cohort 185 cases and 389 controls.	*CYP19*	rs11070843	G/A	23%/15%	OR = 0.6	0.027
	McAninch et al. 2018 [66]	Cohort of 3054 participants from multiple studies	*DIO2*	Thr92AlaD2 (rs225014)	C/T	46%/34%	OR = 1.3 #	0.008 #
AD/Dementia GWAS (excluding *APOE* region)	Reitz et al. 2013 [67]	1968 AD cases and 3928 controls	*ABCA7*	rs115550680	G/A	7%/0%	OR = 1.79	2.2 × 10^−9^
	Kunkle et al. 2021 [68]	2784 AD cases and 5222 controls	*IGF1R/ARRDC4*	rs570487962	C/A	0.01%/0%	OR= 0.10	1.6 × 10^−9^
	Sherva et al. 2022 [69]	4012 ADRD cases and 18,435 controls meta-analyzed with 6641 Proxy cases and 45,970 proxy controls	*ROBO1*	rs11919682	C/G	30%/26%	Dir = −	1.63 × 10^−8^
			*RP11-340A13.2*	rs148433063	T/C	97%/100%	Dir = −	8.65 × 10^−9^
		Above meta-analyzed with Kunkle et al. 2021 [68] cases/controls	*TREM2/TREML2*	rs73427293	A/T	86%/100%	Dir = −	2.95 × 10^−9^
			*RP11-157D6.1 /CD2AP*	rs7738720	T/C	10%/0%	Dir = −	1.14 × 10^−9^
			*ABCA7*	rs73505251	A/T	15%/0%	Dir = +	3.26 × 10^−10^
	Mez et al. 2017 [70]	AD liability model for 1825 AD cases and 3784 controls	*COBL*	rs112404845	T/A	1.2%/0%	β = 0.47	3.8 × 10^−8^
			*SLC10A2*	rs16961023	G/C	2.0%/0%	β = 0.41	4.6 × 10^−8^
Candidate gene/Brain MRI Traits	Cuenco et al. 2011 [71]	158 Discordant sib pairs	*TTR*	rs3764476/ medial temporal atrophy	A/C	35%/34%	NA	0.069
				rs3794884/medial temporal atrophy	A/C	63%/66%	NA	0.075
Brain MRI Trait GWAS	Melville et al. 2012 [72]	Family cohort including 188 cases and 231 controls	*F5/SELP*	rs3917854/ hippocampal volume	C/T	94%/69%	Dir = +	5.0 × 10^−6^
			*PICALM*	rs17148741/ hippocampal volume	C/T	82%/97%	Dir = −	9.39 × 10^−5^
			*SYNPR*	rs935793/cerebral volume	A/G	79%/80%	Dir = +	7.12 × 10^−5^
*APOE*-region effect modifying SNPs	Choi et al. 2019 [55]	Cohort of 1523 AD cases and 3462 controls	*APOE*	rs405509	T/G	24%/48%	Large effect in EUR but rare in AA; may explain population differences in ε4 effect	
	Le Guen et al. 2022 [54]	Discovery cohort of 2888 cases and 4957 controls	*APOE*	R145C (rs769455)	T/C	2.5%/0%	OR = 3.01 among ε3/ε4 persons	6.0 × 10^−6^
Targeted AD Gene Sequencing	N’Songo et al. 2017 [73]	131 cases and 107 controls/Replication cohort of 67 cases and 233 controls	*PSEN1*	Ser167Phe (rs777923890)	T/C	NA	Alternate allele not present in controls	
			*PSEN2*	Phe111Leu	C/T	NA		
	Cukier et al. 2016 [74]	Variant discovery in 77 cases, Association in 531 cases and 527 controls/Replication cohort of 447 cases and 880 controls	*ABCA7*	rs142076058	14 bp deletion	6.7%/0%	OR = 2.13 (Discovery) OR = 1.65 (Replication)	0.0002 (Discovery) 0.012 (Replication)
	Jin et al. 2015 [75]	906 cases and 2487 controls	*TREM2*	rs2234256	G/A	15%/0%	OR = 1.27	0.01
				rs2234258	T/C	5%/0%	OR = 1.35	0.08
	Logue et al. 2018 [76]	Discovery cohort of 489 cases and 472 controls/Replication cohort of 484 cases and 484 controls	*ABCA7*	rs567222111	11 bp LOF deletion	0.99%/0%	OR = 2.42	0.022
Whole exome sequencing	N’Songo et al. 2017 [77]	WES of 131 AD cases and 107 controls/ Combined discovery + Validation cohort of 198 cases and 538 controls	*ABCA7*	rs3764647	G/A	26%/3.6%	OR = 2.0/ OR = 1.41	0.0019/0.017
				rs3752239	C/A	0.2%/4.1%	OR = ^/ OR = 4.06	0.035/0.044
	Logue et al. 2014 [78]	Variant discovery WES of 7 cases and association/replication cohorts, including 1459 cases and 2263 controls	*AKAP9*	rs144662445	G/A	0.5%/0%	OR = 2.75	0.014/0.0022
				rs149979685	T/C	0.5%/0%	OR = 3.61	0.037/0.0022

* Allele frequencies for 1000 Genomes AFR (African ancestry) and EUR (European ancestry) reference populations, respectively. # Additive model results from meta-analysis. ^ Effect size not reported; Dir = direction of effect indicated as protective (−) or risk (+). NA = Not reported or not applicable.

A subsequent study showed that variants in the sortilin-related VPS10 domain-containing receptor 1 (*SORCS1*) were associated with AD in the same sample, although the evidence was more compelling in the EUR cohorts [63]. However, a nominally significant (*p* < 0.05) association between AD and a 3-SNP *SORCS1* haplotype was observed in the AA cohort. One of the SNPs from that haplotype, rs1887635, was significantly associated with AD in the Caribbean-Hispanic cohort (*p* = 0.001). Vardarajan et al. [64] examined associations with SNPs in other genes involved in vesicular trafficking, particularly those that encode retromer and retromer-associated proteins, in EUR (8309 AD cases, 7366 controls) and AA (513 AD cases, 504 controls) cohorts assembled by the Alzheimer’s Disease Genetics Consortium (ADGC). They identified a nominally significant association of AD with SNPs in seven retromer genes in the EUR sample, of which three (*KIAA1033, RAB7A, SNX1*) were also significant in gene-based tests. Examination of these three genes in the AA dataset revealed no significant SNP associations in *RAB7A* or *SNX1*; however, nominally significant associations were observed with 10 SNPs in *KIAA1033*. Interestingly, these SNPs are distinct from other *KIAA1033* SNPs showing association in the EUR dataset. Further studies are required to determine whether there are population-specific AD-related functional variants in *KIAA1033*.

Suggestive evidence for association with AD in the AA population has also been reported for two hormone-related genes. Reduced risk of AD (OR~0.6) was observed for a pair of variants in *CYP19*, a gene that codes for a protein involved in androgen processing, in the AA subset of a multi-ancestry cohort of females [65]. Another study found that the *DIO2* Thr92AlaD2 (rs225014) missense variant, which is about 10% more frequent in African-ancestry populations compared to EUR populations, was associated with AD in a sample of 3054 AA individuals but not in a much larger group of 9304 EUR individuals [66]. *DIO2* is involved in the activation of thyroid hormone, which has been linked to cognitive dysfunction [79].

Because many of these candidate gene associations have not been observed in AD GWAS focused on AA individuals with much larger sample sizes, it is unclear whether some results are false positives or reveal study design biases, including subject ascertainment. Alternatively, they may reflect small effect sizes or allelic heterogeneity that would require extremely large samples to detect in GWAS.

## 5. Genome-Wide Association Studies for AD Risk

**Figure 1:**
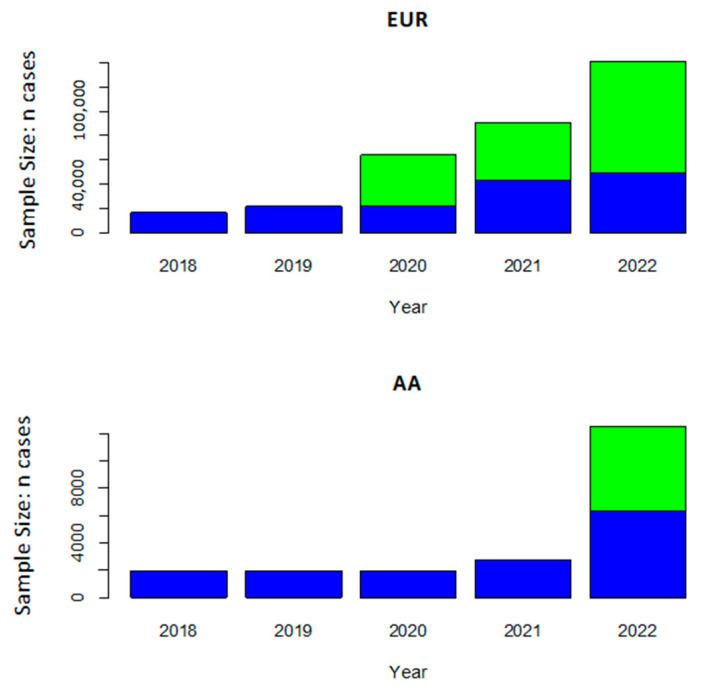
Discovery cohort sample sizes for AD cases in the largest European ancestry and African American AD GWAS by year. The number of cases in the AD case-control cohort GWAS is indicated in blue, and the number of proxy cases (i.e., AD status in the parents) is indicated in green. Although the sample size is much smaller for African American compared to European ancestry GWAS, the size of African American GWAS surged after the publication of a recent study [69].

GWAS has yielded relatively few GWS (*p* < 5.0 × 10^−8^) AD loci in AA cohorts, presumably because the sample sizes of these studies were much smaller than GWAS of EUR cohorts (Figure 1). The discovery samples in the first two published AD GWAS conducted in AA cohorts included fewer than 1300 AD cases and 1000 controls [47,80]. Neither study identified GWS associations with variants outside of the *APOE* region. However, a meta-analysis of the top-ranked results from the discovery sample and a much larger replication sample in the Kamboh et al. GWAS (4013 cases and 4274 controls) yielded suggestive associations (*p* < 1 × 10^−4^) with SNPs in two AD loci established in EUR cohorts (*BIN1* and *PICALM*) and association with a novel locus (*PPP1R3B*) [80]. The first GWS association outside of the *APOE* region from an AA GWAS was identified with rs115550680 in *ABCA7* in an ADGC dataset that contained 1968 AD cases and 3928 controls [69], including the subjects from prior GWAS [47,80]. This SNP is in weak LD with the peak *ABCA7* SNP reported in the large GWAS conducted by Bellenguez et al. [33] in EUR cohorts (rs12151021, D′ = 1 and R2 = 0.053 in the 1KG AFR cohort). Notably, the effect size of the rs115550680 AD risk allele (OR = 1.79) approached the effect of the SNP defining the *APOE* ε4 isoform (rs429358, OR = 2.31). Gene-based aggregated variant tests implicated several other known AD risk genes, including *BIN1, EPHA1, CR1*, and *CD33*.

Many genes will escape detection in association studies because AD is a complex phenotype, and signals for genes that influence specific mechanisms are masked by influences of other genetic and non-genetic risk factors. To address this concern, Mez et al. developed a quantitative AD posterior-liability score of AD status controlling for *APOE, ABCA7* SNP rs115550680, age, sex, and known other AD risk factors (including education, smoking, and diabetes) and the prevalence of AD given these risk factors [70]. A GWAS of this liability score in an AA cohort of 1825 AD cases and 3784 cognitively normal controls identified GWS evidence for association with two novel loci: rs112404845 (*p* = 3.8 × 10^−8^) upstream of *COBL* and rs16961023 (*p* = 4.6 × 10^−8^) downstream of *SLC10A2*.

More recently, additional GWS and suggestive associations have been identified in larger AA samples. An ADGC GWAS by Kunkle et al. [68] that included 2784 AA AD cases and 5222 controls did not observe GWS associations with any common variants outside of the *APOE* region (including the *ABCA7* rs115550680 SNP), although suggestive evidence of association (*p* < 0.05 × 10^−7^) was obtained for common variants in *ALCAM, EDEM1, GPC6*, and *VRK3*, as well as for a rare variant in *RBFOX1*. GWS association was also found for a rare chromosome 15 intergenic variant (rs570487962) using a model which adjusted for *APOE* ε4 dosage. Suggestive gene-based associations (*p* < 10^−4^) were observed for *STARD10* and *ARAP11*. Follow-up analyses in autopsied EUR AD cases and controls from the ROSMAP study [81] showed that *ALCAM, ARAP1, GPC6*, and *RBFOX1* expression in the brain was associated with amyloid load, and *STARD10* expression was associated with Tau pathology.

Sherva et al. [69] conducted a larger-scale AA dementia GWAS using a cohort from the Million Veteran Program (MVP) [82], a US Department of Veterans Affairs (VA) funded initiative that has generated genetic data linked to electronic medical record (EMR) data and survey responses [82] from more than 650,000 veterans to date [83]. Because of difficulties in discriminating AD from other forms of dementia (e.g., vascular, Lewy body, and frontotemporal dementias) based on EMR data, these investigators broadened the outcome to AD and related dementias (ADRD). An AA sample including 4012 ADRD cases and 18,435 controls was ascertained from the MVP cohort using International Classification of Diseases (ICD) codes. In addition, a GWAS for “proxy” dementia, defined as self-reported AD/dementia in the parents of the MVP participants, was performed in a manner analogous to proxy AD GWAS using EUR participants in the UK Biobank [31,32,33,34]. Analyses were performed separately for 4385 proxy maternal cases and 2256 proxy paternal cases, each compared with 45,970 controls. Results from the two AA proxy GWAS and the ADRD AA GWAS were combined by meta-analysis. GWS associations were found with many variants in the *APOE* region and with SNPs at two novel loci: rs11919682 near *ROBO1* (*p* = 1.63 × 10^−8^) and rs148433063 near *RP11-340A13.2* (*p* = 8.56 × 10^−9^). Sensitivity analysis with a more restrictive AD phenotype indicated that the associations are likely specific to AD rather than other forms of dementia. To obtain results from the largest possible sample, Sherva et al. combined results from the AD/proxy AD MVP GWAS with those from the Kunkle et al. AA AD GWAS [68]. GWS associations were obtained for several known AD risk loci, including *ABCA7* (rs73505251, *p* = 3.26 × 10^−10^), *TREM2* (rs73427293, *p* = 2.95 × 10^−9^), and *CD2AP* (rs7738720, *p* = 1.14 × 10^−9^). It is interesting that these peak SNPs differ from those observed in studies of EUR subjects and other AA cohorts. A suggestive association was also observed with several rare variants, including rs509334, which is located upstream of *SORL1*, a known AD risk gene [33,62].

## 6. AD Biomarker Studies

The development of AD biomarkers, especially ones that can identify prodromal indications of AD pathogenesis, is important and a major activity in academic and commercial laboratories. In particular, many efforts have focused on measures of amyloid-β (Aβ) and Tau in cerebrospinal (CSF) fluid [84] and peripheral blood [85], as well as the development of sensitive assays for neurofilament light chain (NFL) and glial fibrillary acidic protein (GFAP) in blood [85], and improvements in positron emission tomography (PET) scanning protocols for detection of Aβ and Tau accumulation in the brain [86]. However, recent work has indicated that baseline levels of some of these biomarkers and that the relationship between biomarker changes and symptomatology may differ between AA and EUR individuals [87,88]. Because some of the differences in the relationship between biomarkers and AD symptomatology may be due to a genetic effect, cross-ancestry biomarker studies are warranted. However, genetic studies of these biomarkers and other endophenotypes have so far primarily been performed using EUR cohorts (e.g., [89,90,91,92,93,94,95,96]), including studies that have relatively few AD cases (e.g., [97,98,99,100]). To date, there have been few published genetic association analyses of AD biomarkers in AA AD cohorts. A candidate gene study conducted in 158 AA and 469 EUR discordant sibships that focused on the transthyretin (*TTR*) gene identified a significant association of an MRI measure of medial temporal atrophy with several SNPs and a 3 SNP haplotype in the EUR families [71]. Although there were no significant associations in the AA sibships likely due to the three-fold smaller sample, two SNPs (rs3764476 and rs3794884) were nearly significant (0.05 < *p* < 0.08) with effect directions matching those in the EUR sibships. A subsequent GWAS of MRI traits conducted in an enlarged sample of MIRAGE AA and EUR families, as well as ADNI participants, identified GWS associations for hippocampal volume (HV) with SNPs in the *APOE, F5/SELP, LHFP*, and *GCFC2* regions [72]. These associations were supported by evidence in each dataset, including the AA families. The most significant associations in the AA families were observed for HV with rs3917854 near *F5* and *SELP* (*p* = 5.0 × 10^−6^) and rs17148741 in *PICALM* (*p* = 9.39 × 10^−5^) and for cerebral volume with rs935793 in *SYNPR* (*p* = 7.12 × 10^−5^). A recent multi-ancestry GWAS of PET-amyloid imaging included an African-ancestry cohort (*n* = 359) [101]. While most of the loci implicated in the joint analysis were likely accounted for the much larger EUR cohort (*n* > 11,000), the African-ancestry cohort analysis yielded one GWS association with rs2271774 near *PTDS1* (*p* = 2.25 × 10^−9^).

## 7. Sequencing Studies in African American Cohorts

Many studies have used targeted sequencing of candidate genes, WES, or whole genome sequencing (WGS) to identify new AD risk loci or causal variants in established AD loci. Because of the larger expense for WGS compared to other sequencing approaches, coupled with the low power to detect rare variant associations in modest-size samples, many sequencing studies of AD in AA cohorts have focused on genes that were previously implicated in studies performed in larger EUR cohorts. However, greater use of WGS in studies of African ancestry populations is anticipated because of the recent rapid decline in sequencing costs and improvements in sequencing technology, as is already underway for the ADSP [102].

Sequencing studies have identified rare African ancestry-specific *PSEN1* and *APP* mutations for early-onset AD [103,104,105,106]. Motivated by reports of association of variants in these genes with late-onset AD in EUR cohorts [107,108], N’Songo et al. sequenced the coding regions of *PSEN1*, *PSEN2*, and *APP* in an AA sample of 131 late-onset AD cases and 107 controls [73]. They identified 14 rare variants predicted to have a functional impact, including 6 that were observed only in AD cases. These 6 variants were examined in a replication AA sample of 67 AD cases and 233 controls, and two (*PSEN2*: NM_000447: Phe111Leu and PSEN1: rs777923890) were not observed in the controls, suggesting that they may contribute to late-onset AD risk.

**Figure 2:**
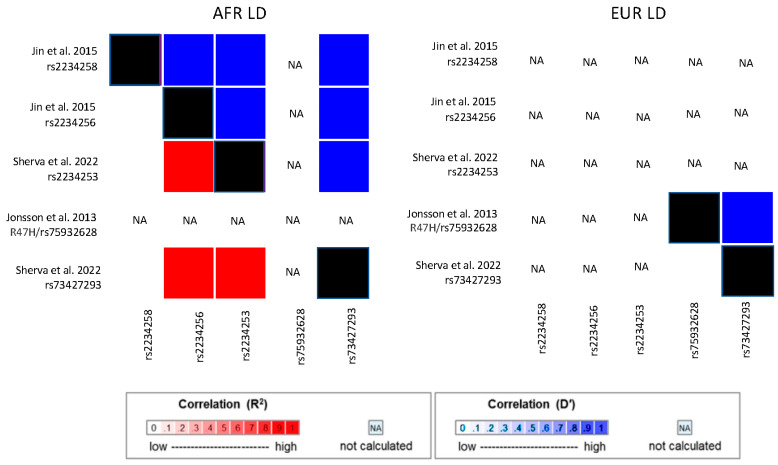
Linkage disequilibrium (LD) among *TREM2* variants associated with AD risk in African American cohorts and the R47H variant associated with AD in European ancestry cohorts [35,69,75]. LD presented for both African-ancestry (AFR) and European ancestry (EUR) populations from the 1000 Genomes reference panel. NA indicates that LD was not estimated because one or both variants were too rare or absent in that population. Values on the diagonals which are necessarily 1.0 are shaded in black.

**Figure 3:**
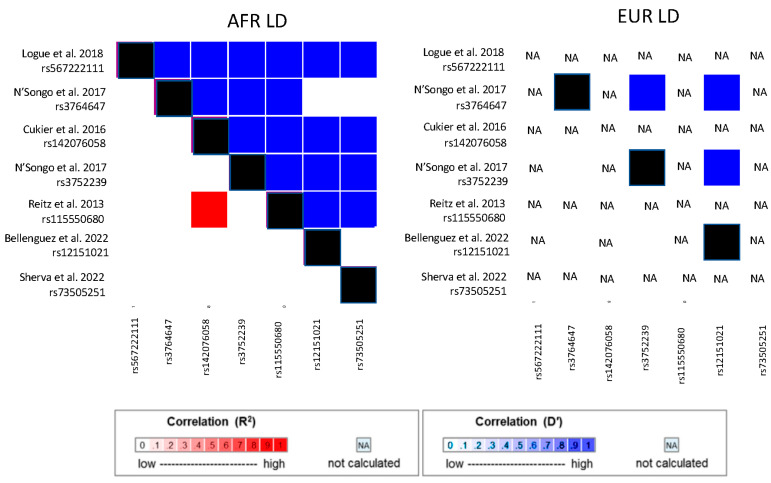
Linkage disequilibrium (LD) of *ABCA7* variants associated with AD risk in African American cohorts and the peak AD SNP (rs12151021) associated with AD in European ancestry cohorts [33,67,69,74,76,77]. LD presented for both African-ancestry (AFR) and European ancestry (EUR) populations from the 1000 Genomes reference panel. NA indicates that LD was not estimated because one or both of the variants were too rare or absent in that population. Values on the diagonals which are necessarily 1.0 are shaded in black.

Potentially functional AD-related variants in *TREM2* and *ABCA7* were identified by targeted gene sequencing studies of AA cohorts. Cukier et al. [74] sequenced 77 subjects who have the intronic *ABCA7* risk allele rs115550680, which was previously associated with AD risk in an ADGC AA cohort [67]. They identified a 44 bp deletion that is common in African ancestry populations (rs142076058), in strong LD with rs115550680, and associated with AD in two independent cohorts [69]. Sequencing of *TREM2* in a pooled AA cohort of 202 AD cases and 136 controls and an independent AA cohort of 179 AD cases and 334 controls identified 6 coding variants that were subsequently genotyped and evaluated for association in an AA sample containing 906 cases and 2487 controls [75]. Although none of the findings were significant, the top-ranked variants, one likely to be benign (rs2234256, OR = 1.27, *p* = 0.01) and rs2234258, which was predicted to impact the short *TREM2* isoform (OR = 1.35, *p* = 0.08), were significantly associated with AD/ADRD in the MVP GWAS (rs2234256, *p* = 4.64 × 10^−9^ and rs2234258, *p* = 5.48 × 10^−7^) [69] and are in high LD with each other and the peak *TREM2* SNPs from the Kunkle et al. GWAS (rs73427293 and rs2234253; Figure 2). The *TREM2* R47H missense variant (rs75932628) is rare in EUR populations (MAF = 0.005 in 1000 genomes EUR cohort) and is absent in African-ancestry populations (not observed in the 1000 genomes AFR cohort). Hence, rs75932628 is unlikely to underly the *TREM2* associations observed in AA cohorts [75] or in a Caribbean Hispanic cohort that has substantial African ancestry [109]. Logue et al. sequenced coding and flanking proximate regions of approximately 100 genes associated with AD and AD-related traits in an AA discovery cohort of 489 AD cases and 472 controls [76]. They identified an association of AD with an 11 bp loss-of-function deletion in *ABCA7* (OR = 3.57, *p* = 0.038), a finding which was bolstered by the inclusion of data from an AA replication cohort of 484 cases and 484 controls (*p* = 0.022). A low correlation between the deletion and other AD-associated *ABCA7* variants indicates that there are likely multiple independent AA AD risk loci in *ABCA7* (Figure 3).

A WES study conducted in a relatively small AA sample of 131 AD cases and 107 controls identified a nominally significant association of AD with *ABCA7* missense variants rs3764647 and rs3752239 [77]. Aggregated rare variant tests also found nominally significant associations with several known AD genes, including *ABCA7, MS4A6A*, *PTK2B*, and *ZCWPW1* [77]. In contrast, a very small WES study of seven AA AD cases (including two affected sib pairs) from the MIRAGE Study identified two rare missense mutations (rs144662445 and rs149979685) in *AKAP9*, a gene not previously associated with AD, that were present in more than one sequenced subject with AD [78]. Subsequent genotyping of these variants in the entire MIRAGE AA cohort of 422 cases and 394 controls and in an ADGC AA cohort of 1037 AD cases and 1869 controls revealed that they are in high LD and significantly associated with AD in each cohort: MIRAGE rs14466245, *p* = 0.037 and rs149979685, *p* = 0.014); ADGC: *p* = 0.0022 for both variants. Examination of African-ancestry haplotypes in 1000 Genomes Project [110] data indicated that these variants likely functionally impact the encoded Akap9 protein. A subsequent WGS study identified another rare *AKAP9* coding mutation (p.R434W) that segregated in two large Caribbean Hispanic families and was nominally associated with a five-fold increased risk of AD [109]. No WGS studies of AD with a major focus on AAs have appeared in the literature at the time of this writing.

## 8. Linking AKAP9 to AD by Functional Studies

*AKAP9* encodes a member of a family of proteins that bind signaling molecules to their targets [111]. The rs144662445 variant is located in the protein kinase alpha (PKA) binding subunit of *AKAP9* [78,112]. This suggests an important potential risk mechanism, as activated PKA leads to phosphorylated Tau, which is a hallmark of AD neuropathology. To test this hypothesis, Ikezu et al. examined phosphorylated Tau and amyloid precursor protein (APP) levels in lymphoblastoid cell lines (LCLs) from 11 AA subjects with at least one of these variants (AKAP9+) and 17 AA subjects lacking these mutations (AKAP9-) [113]. LCLs were transduced by viral vectors expressing causative AD mutations in APP or human full-length wild-type Tau. Cell lysates were analyzed for total APP, Aβ40, and total and T181 phospho-Tau (pTau). *AKAP9* mutations had no effect on Aβ40/APP but significantly increased pTau/Tau ratio in LCLs treated with phosphodiesterase-4 inhibitor rolipram, which activates protein kinase A. This study showed the impact of rare functional *AKAP9* mutations on Tau, a central mechanism of AD pathogenesis, in LCLs derived from AD and control subjects.

To further the understanding of how the *AKAP9* mutations influence Tau phosphorylation, You et al. performed a proteomic analysis of the Tau interactome using SH-SY5YP301L neuronal cells, which were CRISPR-Cas9-engineered to carry the *AKAP9* rs144662445 mutation [114]. Their analyses revealed an enrichment of RNA binding proteins and a decrease of proteasomal molecules in rolipram-treated cells with the *AKAP9* mutation. Those cells also displayed increased Tau phosphorylation at the S396 and S404 sites and increased oxidative stress. These changes in the Tau interactome mirrored disruptions noted in AD brain tissue vs. controls, and oxidative stress has been shown to play a role in neuronal dysfunction in AD brains [115,116].

## 9. Polygenic Risk Scores

One of the advances powered by large-scale GWAS is the development of methods for computing polygenic risk scores (PRSs) and using them to understand the shared genetic risk for multiple diseases and biological processes. A PRS summarizes an individual’s genetic risk for a particular disease or related trait based on GWAS summary results. They are computed as a weighted sum of the coefficient (effect size estimate) for each SNP generated from a GWAS, which is typically the log OR of the per-allele risk for binary disease outcomes. In EUR cohorts, these scores have been shown to predict AD risk better than *APOE* genotype and demographic risk factors alone [117]. AD PRSs have been associated with AD biomarkers [118] and neuroimaging traits [119,120,121]. The AD PRS has potential promise to distinguish early-stage AD among persons with mild cognitive impairment many years before AD symptoms typically appear [122]. However, the performance of a PRS is greatly influenced by the sample size of the GWAS used to determine the weights ascribed to the SNPs included in the PRS calculation [123]. Moreover, the strength of an association between a PRS and a trait depends on the correspondence between the effect sizes of the constituent SNPs in the GWAS and in the target sample, and the SNP effect sizes in AA cohorts will usually differ from those derived in EUR cohorts. Indeed, the accuracy of a PRS is reduced when there is a mismatch between the ancestry of the cohort used in the GWAS and the target cohort to which the PRS is applied [124,125,126]. However, there is some evidence that including multiple ancestry risk scores can improve PRS performance relative to using smaller-sample size individual ancestry GWAS results for PRS calculation [127]. Therefore, increasing sample sizes of AA cohorts and cohorts for other non-EUR populations in genetic studies has been identified as a priority for genetic research [124] as a means to more accurately determine genetic risk and to develop population-specific therapeutic strategies and subsequently reduce the growing health gap between populations.

## 10. Leveraging African American Cohorts to Increase Genetic Discovery

In addition to improving the accuracy of risk prediction for AA individuals, genetic studies conducted in AA cohorts have great potential to generate new and important insights into AD pathogenesis. Several studies have demonstrated that non-EUR cohorts can have a much greater impact relative to their sample size. For instance, a GWAS conducted by the PAIGE Study for multiple traits in 49,839 non-EUR individuals (a relatively small sample compared to GWAS for analogous traits in EUR cohorts) found 27 novel trait associations and secondary associations at 38 known trait loci and replicated over 1000 EUR GWAS associations [128]. African ancestry populations have more diverse genomes than EUR and EASN populations because African ancestry populations have evolved over a much longer period of time than other continental populations that were founded by small bands migrating from Africa. Consequently, the number of rare variants observed in any gene or other portion of the genome will be greater in African-ancestry cohorts than in EUR or EASN cohorts. There is a growing understanding that this greater genetic diversity can yield important insights into pathways to AD that would be missed by focusing on Europeans only. This is illustrated by a multi-population AD GWAS that included a large EUR sample and smaller groups of AA, EASN, and Israeli-Arabs that identified novel GWS associations with rs11168036 located between *PFDN1* and *HBEGF* (*p* = 7 × 10^−9^), rs7920721 located between *USP6NL* and *ECHDC3* (*p* = 3 × 10^−8^), and *BZRAP1-AS1* SNP rs2632516 (*p* = 4 × 10^−8^) [21]. Analyses of models conditioned on the top SNP at the *PFDN1/HBEGF, USP6NL/ECHDC3,* and *BZRAP1-AS1* loci confirmed a single association signal in each region. These GWS associations, except for rs7920721, were supported by evidence in multiple ethnic groups.

## 11. Importance of Racial/Ethnic Identity and Social Determinants of Health in Genetic Studies of AD

As discussed briefly in the Introduction, race is a social construct that is viewed as having little scientific value in representing distinct ancestral populations [15,16]. Therefore, many genetic studies focus on ancestry determined by clustering of genetic profiles relative to reference populations rather than on self-identified race or ethnicity. However, simply replacing ancestry, especially continental ancestry, for race does not accurately represent the diversity of human populations or the continuous nature of human genetic variation [17]. Moreover, both of these methods for grouping individuals are correlated with SDOH in similar but not identical ways [18]. Therefore, focusing on ancestral categorizations, scientists risk genetic essentialist framing of complex influences on the development of AD. Jointly considering race, concomitant social variables, and ancestry may be one way to incorporate a deeper understanding of the ways in which environmental pressures, such as poorer education and barriers to good nutrition, are genetically mediated [16,129]. However, care must be taken to ensure the use of race is appropriately contextualized as a proxy for SDOH until the more proximal factors themselves can be disentangled from race. The picture may be further muddled by the practice of race norming of assessments of patient cognition, which is incorporated by default into some tools that evaluate dementia severity, for instance, through the use of Heaton norms [130]. Race-norming attempts to compare patients within a particular group rather than across groups, but, in dementia assessment, can pathologize race, as AA individuals are compared against a lower baseline level of cognition than EUR individuals [131,132]. This can lead to differences in the type and severity of dementias between AA- and EUR-study cohorts and obscure systemic and environmental factors that lead to disparities across groups [133], thereby distorting study conclusions. Consideration of the interaction of SDOH and genetic factors is a complex but important endeavor in moving towards precision health. 

Genetic research involving AA individuals should be undertaken with several interrelated factors in mind. As articulated by the National Academies committee “Use of Race, Ethnicity, and Ancestry as Population Descriptors in Genomics Research,” the foundational principles to the inclusive practice of genomics research are respect, equity and justice, beneficence, validity and reproducibility, transparency, and replicability [134]. Research must be performed and communicated in a way that promotes health without amplifying barriers to opportunities, exemplified by concerns surrounding genetic testing for sickle cell disease and carrier status [135]. To achieve this goal, scientists must not only simply include samples from diverse ancestral populations in large-scale genomic studies but truly partner with communities that have been historically excluded from research to ensure equitable benefit sharing [136,137] and assemble diverse teams to support these partnerships [138]. With this motivation, the ADSP prioritized the inclusion of diverse populations [139] and partnership with local investigators in many parts of the world, including the Caribbean, South America, Africa, Asia, and the Middle East, to enroll diverse cohorts that are sufficiently powered for genetic association studies [55,102,109,140,141,142,143]. In addition, while accurate risk prediction which takes into account SDOH and ancestry is important, and indeed this is one of the primary motivations of the priority to increase non-EUR representation in genetic studies, ancestry-specific protocols for treatment or identification of those at risk should be carefully evaluated and applied in a way that is respectful and does not limit access to care [16]. Identification of risk factors and risk markers in specific populations should not be the end goal but rather a way to refine hypotheses about the ways in which genetic, environmental, and cultural factors combine to influence health disparities. In summary, AD genetic research should be presented in a manner that avoids typological thinking promoting genetic determinism, stresses the impact of environmental and health factors, clearly indicates that the late-onset AD variants are neither necessary nor sufficient to cause AD [144,145], and promotes equitable benefits of research.

## 12. Conclusions

Increasing sample sizes of AA cohorts is and should remain a priority in genetic studies of AD and dementia. The studies highlighted in this review demonstrate the value of AA AD studies to increase our knowledge about the genetic underpinnings of AD risk. These studies will increase our understanding of the genetic architecture of AD in the AA population, help minimize health gaps that might develop because of the implementation of precision medicine approaches for AD, and lead to more accurate genetic testing and estimates of dementia risk. In elevating these goals, it is essential that we take care to be attentive to the distinctions between race and ancestry, avoid the use of harmful language and labels that reify notions of racial hierarchy, and form true partnerships between communities of participants and scientists to ensure identification of equitable, shared goals.

## Figures and Tables

**Table 1:** Odds ratios (OR) or relative risk (RR) for AD for individual or aggregated APOE genotypes compared to the ε3/ε3 genotype among African American cohorts.

Study	Cohort Characteristics	*APOE* Genotype								
		ε2/ε2 ε2/ε3 ε2/ε4	ε2/ε2 ε2/ε3	ε2/ε2	ε2/ε3	ε2/ε4	ε3/ε4	ε2/ε4 ε3/ε4	ε2/ε4 ε3/ε4 ε4ε/4	ε4/ε4
Tang et al. 1996 [45]	New York community-based cohort of 106 AD cases and 154 controls	RR = 1.3 (*p* > 0.05)	.	.	.	.	.	RR = 0.6 (<0.05)	.	RR = 3.0 (<0.05)
Farrer et al. 1997 [29]	Multi-center Cohort of 235 cases and 240 controls	.	.	OR = 2.4 (>0.05)	OR = 0.6 (>0.05)	OR = 1.2 (>0.05)	OR = 2.7 (<0.05)	.	.	OR = 12.5 (<0.05)
Sahota et al. 1997 [46]	Indianapolis community-based cohort of 60 AD cases and 228 controls	.	.	.	OR = 0.43 (>0.05)	OR = 0.34 (>0.05)	OR = 1.20 (>0.05)	.	.	OR = 4.83 (<0.05)
Tang et al. 1998 [43]	New York community-based cohort of 48 incident cases and 128 controls	.	.	.	.	.	.	.	RR = 1.0 (*p* > 0.05)	.
Graff-Radford et al. 2002 [44]	338 cases, 301 unrelated controls, and 108 siblings from the MIRAGE Study	.	.	.	OR = 0.41 (*p* < 0.05)	.	OR = 2.6 (*p* < 0.05)	.	.	OR = 10.5 (*p* < 0.05)
Murell et al. 2006 [48]	Indianapolis community-based cohort of 162 AD cases and 318 controls	.	OR = 0.42 (0.02)	.	OR = 0.46 (0.047)	OR = 1.34 (0.61)	OR = 2.32 (<0.001)	.	.	OR = 7.19 (<0.001)
Logue et al. 2011 [47]	513 Cases and 496 controls from the MIRAGE and GenerAAtions Studies	.	OR = 0.43 (0.0094)	.	.	.	.	OR = 2.08 (2 × 10^−7^)	.	OR = 2.62 (3 × 10^−14^)

*p*-values indicated in parentheses; . = not calculated or not reported.

## Data Availability

Not applicable.
